# Safety Program Elements in the Construction Industry: The Case of Iraq

**DOI:** 10.3390/ijerph18020411

**Published:** 2021-01-07

**Authors:** Mohanad Kamil Buniya, Idris Othman, Serdar Durdyev, Riza Yosia Sunindijo, Syuhaida Ismail, Ahmed Farouk Kineber

**Affiliations:** 1 Department of Civil & Environmental Engineering, University Technology PETRONAS, Seri Iskandar 32610, Perak, Malaysia; idris_othman@utp.edu.my (I.O.); Ahmed_17008588@utp.edu.my (A.F.K.); 2 Department of Engineering and Architectural Studies, Ara Institute of Canterbury, Christchurch 8011, New Zealand; durdyevs@ara.ac.nz; 3 Faculty of Built Environment, UNSW Sydney, Sydney, NSW 2052, Australia; r.sunindijo@unsw.edu.au; 4 Razak Faculty of Technology and Informatics, Universiti Teknologi Malaysia, Kuala Lumpur 54100, Selangor, Malaysia; syuhaida.kl@utm.my

**Keywords:** construction industry, Iraq, management commitment, safety program, safety training

## Abstract

The construction industries’ unsafe conditions require increased efforts to improve safety performance to prevent and reduce accident rates. Safety performance in the Iraqi construction industry is notoriously poor. Despite this condition, safety research has so far been neglected. Implementing a safety program is a proven initial step to improve safety. Therefore, the aim of this study is to identify the key elements of a safety program in the Iraqi construction industry. To verify and validate a list of safety program elements identified in the literature review, a mixed method approach was used by using interviews and questionnaire surveys. A final list of 25 elements were then analyzed using exploratory factor analysis. The analysis found that these elements can be grouped into four interrelated dimensions: management commitment and employee involvement, worksite analysis, hazard prevention and control systems, and safety and health training. This study contributes to the body of knowledge on safety in the Iraqi construction sector, a research area which has not been adequately investigated previously. They also help decision-makers focus on key elements that are needed to start improving safety performance in this context.

## 1. Introduction

In spite of the efforts to improve and develop safety performance, the accident rates in the global construction industry are still unacceptably high [1,2]. For example, in 2018, 28% of work fatalities in the US happened in the construction sector, which was the highest proportion among all industrial sectors [3]. Likewise, in 2019, the UK construction industry fatality rate was 1.31 per 100,000 workers, 3 to 1 higher than the industry average [4]. Accounting for 38% of total accidents, the Iraqi construction industry is experiencing the same problem [5]. Despite the implementation of various advanced construction methods and technologies, such as building information modeling [6] and off-site manufacturing [7], there is still a need to improve safety performance in the construction industry, in particular for developing countries, where attention to safety is still severely lacking. This improvement is important because poor safety performance has a negative influence on the achievement of project objectives. Poor safety performance has been attributed to various reasons, and one of the most important, particularly in developing countries, is the lack of safety regulations and their inadequate enforcement [8,9]. This factor is considered a crucial stepping stone for improving safety in the construction sector [10].

From the research side, most of the previous studies were conducted in developed countries. Hallowell and Gambatese [1] use the USA as a case study to identify the effective safety elements to determine a strategy to prevent injuries by quantifying the element’s ability to minimize the risk in construction safety. Moreover, Hill [11] identified the safety program elements to improve safety performance in US construction industry.

Iraq is one of these developing countries suffering from the lack of safety management in the construction industry. The construction industry faces a rapid growth with government support in last few years [12]. According to [13], the accident rate in the Iraq construction industry in 2018 was 38% of overall industrial accidents. Despite the importance of safety in the construction industry, there has been a lack of research that focuses on safety in the Iraqi construction industry. A robust safety program is needed to facilitate safety performance improvements in this context. Although this subject has been studied in other countries [14,15,16], it requires a country-specific diagnosis due to Iraq’s operational and cultural contexts. In addition, there is a lack of research in safety program implementation in the Iraq context [13,17,18]. With this in mind, the present study aims to identify the essential elements of the safety program. The findings of this study can help the top management and stakeholders to implement an effective safety program in the Iraqi construction industry in order to improve safety performance. The study makes a contribution to the construction safety field by addressing the gap in Iraq. As an example from a developing country, the study offers knowledge to inform the construction practitioners in other developing countries to create safer and healthier construction project environments.

## 2. Literature Review

### 2.1. Construction Safety Program

The Oregon Occupational Safety and Health Division used the term “safety and health program” to describe what people can do in the workplace to monitor and control illnesses and injuries [17]. Anton [18] reported that the safety program is applied to monitor the working environment, facilities, procedures, and staff to reduce accidents and fatalities in projects. Rowlinson [19] described that the goal of a safety program implementation in the construction industry is to detect and prevent unsuitable behavior that causes accidents. An effective safety program in construction helps prevent accidents and illnesses from occurring in the workplace [20]. Furthermore, safety programs can reduce costs associated with accidents, reduce absenteeism, increase productivity, and enhance worker morale [21].

The existence of regulation weaknesses and particularly safety management programs in construction projects is behind many on-site safety problems [22]. Poor and ineffective implementation of safety programs in developing countries’ construction industries leads to failure in meeting the needs of modern competitive businesses in the globalized market [23,24]. Compared to developed countries, outdated safety regulations, lack of enforcement, and poor safety awareness are main issues faced by developing countries [25].

Robust safety legislation and regulations and their strong enforcement and support through various safety training sessions have been fairly successful in supporting safety program implementation in developed countries [26,27,28,29,30]. On the contrary, safety rules in developing countries hardly exist, and those that exist are inadequate, ineffectual, and outdated [30]. Additionally, the authorities usually fail to successfully implement these safety rules, and job hazards are either not identified or identified as less dangerous than they are [28,30].

### 2.2. Safety Program Elements

A safety program element is an effective approach to encourage safety in the workplace and provide a mechanism to manage safety risks. Implementing a safety program has been seen as effective to address common safety problems in the construction industry, including lack of safety policy and regulations, lack of safety training, unreasonable project schedule, inadequate safety-related resources, low safety commitment, and poor safety awareness [23,24,25,26,27,28].

Studies have been conducted to identify the key elements of an effective safety program in the construction industry. For example, Alarcón, Acuña [31] identified nine injury prevention strategies, including accident investigation, safety resources, management commitment, safety training, management training, safety rewards, employee involvement, audits and certification and average accident rate. Findley, Smith [32] identified safety program elements for accident prevention and reduced accident costs. They concluded that management commitment, employee involvement, worksite analysis, hazard prevention and control, and training are effective safety program elements in the US construction industry. In previous studies, Hill [11] has identified safety program elements including hazard prevention and control, worksite analysis, and training. Hinze, Devenport [33] identified several elements of a safety program and suggested that the components should be designed to meet a specific purpose for the company, rather than using another company’s existing safety program. Hallowell and Gambatese [1] recognized that identifying effective safety elements can be used to develop a strategy to prevent injuries by quantifying the ability of the elements to minimize safety risks. Table 1 summarizes the effective elements of safety programs that were identified in previous studies.

## 3. Research Method

**Figure 1:**
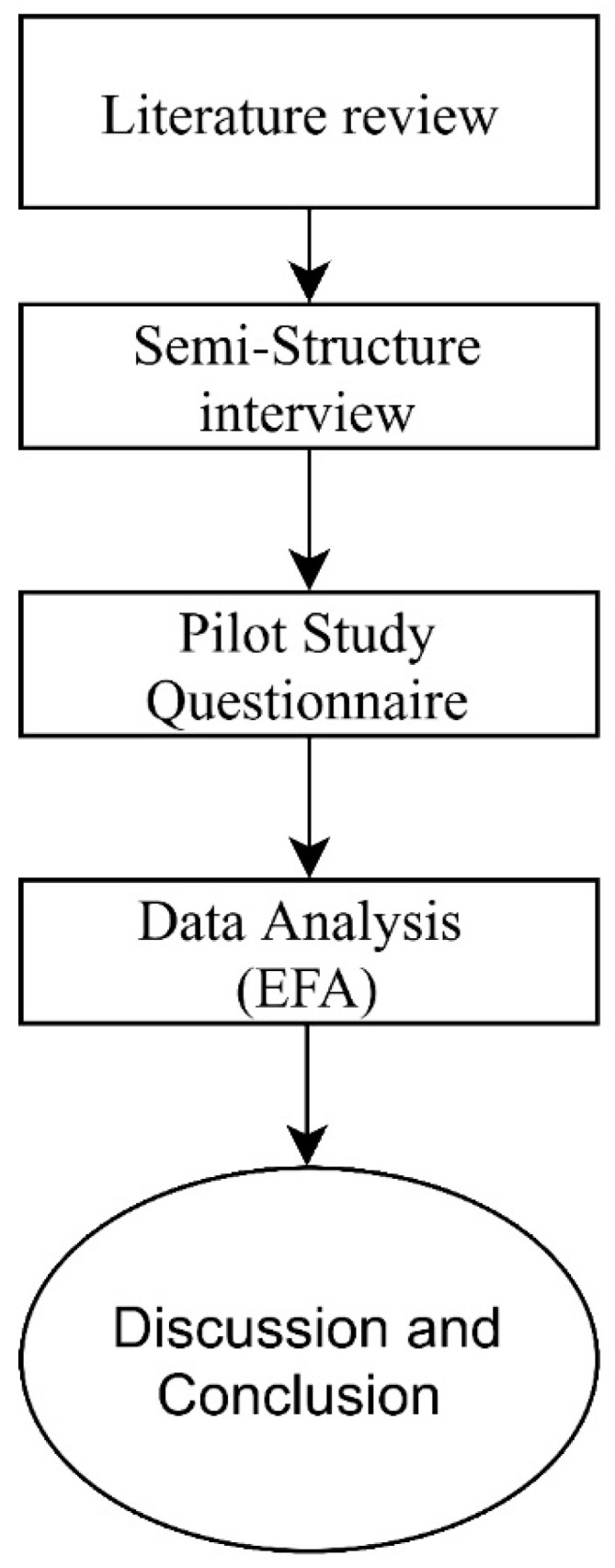
Research flowchart adopted from [49].

This research aims to improve the public health condition of construction projects in the Iraqi construction industry through the implementation of safety programs. The study used a mixed method research. Mixed method research is important since no particular methodology is appropriate for all analysis [47]. It is verified that mixed methods to investigate the same problem will help uncover several recurrent themes or proportionate relationships between elements. Figure 1, used by Buniya, Othman [13,48], shows the study procedures for the research. The research began with a comprehensive analysis of previous studies, then a qualitative study was completed by interviewing 16 experts from the Iraqi construction industry to refine the selected elements from previous studies. The reliability and validity tests were conducted, then several discussions were conducted for various conclusions and recommendations.

### 3.1. Semi-Structured Interviews

The interviews aim to obtain in depth insights on the effects of the safety elements on safety program implementation. According to Sanders [50] and Hesse-Biber [51], ten interviewees can be considered an appropriate number for this kind of study because data saturation is usually achieved. Table 2 shows the sixteen experts who participated in the semi-structured interviews from the construction industry in main cities in Iraq [13,52]. The interviewees were selected based on their experience, education, and work positions. All interviewees had extensive construction industry experience ranging from 8 to 40 years, following the previous studies [53,54]. The participants’ roles were diverse, including senior manager, project manager, site engineer, director, and contractor. The participants work in the private or public sector or as a consultant in independent organizations, while their organizations’ main roles include all key positions within the industry, such as client, consultant, and contractor, thus ensuring rich data from various perspectives.

The interviews typically lasted for 60 min [53,54]. Each interview was transcribed, and the transcripts were then coded and organized into separate folders before being analyzed thematically. During the analysis, the transcripts were checked frequently to identify and organize the key themes. The interviewees were asked to assess whether they considered the elements that were essential to the construction industry in Iraq and their responses included new elements that were not mentioned in the list. Some elements were modified based on interview results. The elements which have been validated were then used in the next stage of the study, which focused on examining the interrelations between the elements and safety program implementation.

Interviewees verified the importance of most of the identified components. Some elements were listed more recurrently than others (safety objectives, safety policy, safety committee, training), indicating their relative importance.

The participants recognized the importance of safety and its impact on productivity, staff morale, project goals, and company credibility. They argued that introducing safety programs is a vital step towards improving safety in the Iraqi construction industry. To successfully implement safety programs, several elements were recommended by the interviewees.

### 3.2. Questionnaire Survey

To categorize the safety program elements confirmed in the semi-structured interview, a questionnaire survey was used. The questionnaire items were designed based on the interview results. The questionnaire has two sections. The first section collects the respondents’ demographic background, while the second section assesses the relevance of safety program elements in Iraq’s construction sector. In the second section, a 5-point Likert scale format was used (Least Effective to Very Effective).

The questionnaire was distributed to professionals working in the Iraqi construction sector. The stratified sampling was chosen in this study because safety is relatively uncommon in Iraq, and to reduce bias when choosing sample cases and to ensure that adequate samples are obtained [55]. More than 250 companies were identified during the screening process, and 225 organizations agreed to participate.

To find the groups of the safety program elements, exploratory factor analysis (EFA) was used in this study. The questionnaire was distributed to 200 respondents and 150 valid responses were obtained, representing a 75% response rate. Tabachnick, Fidell [56] suggested that the sample size for EFA studies should be between 150 and 300; thus, the number of respondents is adequate for EFA, as mentioned by Akintoye [57], Moser and Kalton [58].

### 3.3. Respondent Background

**Figure 2:**
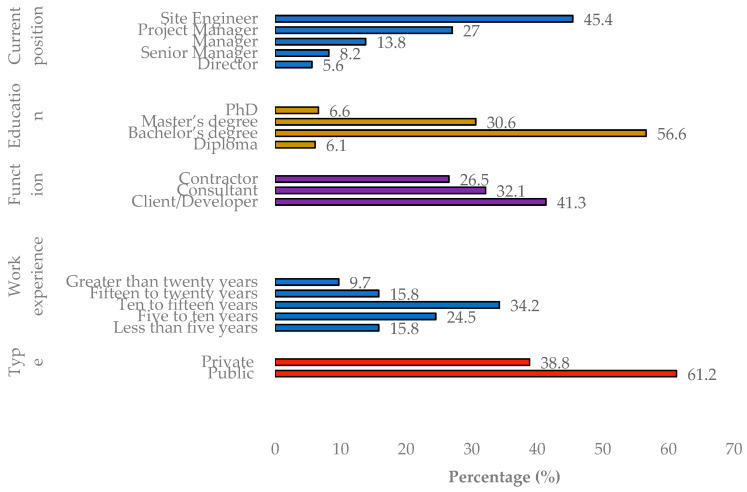
Respondent background rate.

Table 3 and Figure 2 presents the respondent’s demographic information. The work experience in the Iraqi construction industry for 85% of respondents was more than 5 years and more than 60% of them worked in the public sector. This means that the public sector controls and constructed most of the construction projects in Iraq as the majority of the projects were funded by the government. Around 45% of the respondents worked as developers, 29% as contractors and 26% as consultants. The majority of the respondents (64%) worked as project managers and site engineers. The respondents are highly educated, with more than 90% having obtained a higher education degree.

### 3.4. Exploretry Factor Analysis

EFA was conducted to validate the element structure of the 25 safety program elements identified in the earlier steps of this research. EFA is a common analysis technique to identify the relationships between variables and categorize them into groups [59]. The EFA’s objective is to analyze the dimensionality of the groups and improve the interpretation of the loading of factors. It is useful to measure the validity and reliability of the research tool [60]. To maximize the dispersion of loading between factors, the varimax rotation was used in this study. According to Costello and Osborne [61], the varimax is a simple and adequate factor analysis approach and is appropriate to simplify the interpretation of variables.

## 4. Results and Discussion

### 4.1. Exploratory Factor Analysis

The Kaiser–Meyer–Olkin sampling appropriateness measure was 0.863, above the suggested value of 0.6, while the Bartlett sphericity test was important (χ2 (300) = 2588.388, *p* < 0.05). These values indicate that EFA is sufficient for analyzing the data. The diagonals of the anti-image correlation matrix were all over 0.5, encouraging the integration of each item into the analysis. Initial communalities assess the variance in each variable taken into account by all components, and small values (<0.3) indicate variables that do not fit well with the factor solution. In the current analysis, all primary populations were above the average. The factor loadings were above 0.5 (Table 4).

The results of the EFA on the 25 items extracted four factors with an eigenvalue greater than 1. Table 5 shows that the total variance and the eigenvalues that are illustrative of the four themes were 64.54%. The Varimax rotation results showed that the first theme related to hazard prevention and control accounted for 20.622% of the variance and the second theme, management commitment and employee involvement, accounted for 17.85% of the variance. The third component, named worksite analysis, explained 17.424% of the variance, followed by component four, safety and health training, which explained 8.648% of the total variance. Based on the EFA results, two low factor loading elements (HPC.EF8, MCEI.8 and MCEI.EF4) were removed. The Cronbach’s Alpha test was used to test the instrument reliability.

To test the questionnaire’s reliability and measure the construct items’ consistency, the Cronbach’s alpha test was conducted [62]. Table 6 explained the Cronbach’s alpha coefficients of the four components, which range from 0.856 to 0.916, indicating acceptable values. The four components are discussed in the following sections.

### 4.2. Management Commitment and Employee Involvement

Management commitment is a management obligation to engage and maintain behavior towards the achievement of specific objectives [63]. Management commitment is a very important and necessary element to ensure the effective implementation of the safety program and safety efforts [16,38]. Management commitment is also needed to encourage employees to get involved and be proactive in safety program implementation. In this case, employee involvement is manifested in the form of supportive norms towards safety, safety motivation, positive attitudes towards safety, and workers’ active participation in implementing a safety program [16]. This result indicates that the sharing of safety information has positive effects on safety work and an effective situation is important for sharing safety knowledge to encourage safety outcomes.

This component is an underlying component that supports other safety efforts in an organization and significantly impacts safety performance. For example, management can establish an inclusive safety policy that governs safety practices and culture in the organization. The top management should also provide sufficient resources to equip employees and workers with adequate safety knowledge and skills, so that the safety program can be applied and effectively implemented. The top management needs to maintain regular communication with employees at all levels and involve them in safety program implementation. Such commitment and employee involvement makes employees accountable for safety and promotes collaboration to implement the safety program and improve safety performance.

### 4.3. Worksite Analysis

This component is about the identification of hazards and unsafe behavior with the purpose of minimizing and reducing accidents at work. Worksite analysis includes a step-by-step collective evaluation of the workplace to identify actual or potential hazards which may contribute to workplace accidents. Accident investigation reports, violation reports, and other documents of lessons learned can be used to identify hazards and potential behaviors that undermine safety [64]. More importantly, this collective effort requires collaboration between project members to ensure that hazards are identified and safety risks are mitigated effectively when implementing a safety program [35]. Furthermore, worksite analysis should be conducted periodically by inspecting the workplace and assessing work activities to identify new hazards and to ensure that risk management strategies are implemented appropriately to mitigate safety risks.

### 4.4. Hazard Prevention and Control

Construction workers are at risk from a variety of hazards due to changing day-to-day activities. Effective identification of hazards is important to improve safety at work [65]. The prevention and control system can be implemented after identifying hazards and risks in the workplace [1]. Employers should ensure that post-incident procedures and services are in place and/or made available immediately after incidents occurring.

To improve safety performance, the prevention system can be designed to meet the specific cases in each incident; furthermore, the system should be adaptable to emerging risks, as well as to potential changes in the workplace situations.

### 4.5. Safety and Health Training

Training is an essential element of a safety program because it helps to ensure that employees are aware of specific hazards and risks when performing activities in the workplace. Training is also needed to ensure that employees are familiar with relevant safety policies, procedures, and techniques [35]. It is proven that safety performance in the construction sector can be improved by providing effective safety training [16,66]. Due to the dynamic nature of construction work environments, it is important to provide regular training [67,68] so that employees are aware of the changing environments and associated risks. This is important because increased safety awareness, knowledge and skills lead to the successful and effective implementation of the safety program [69].

**Figure 3:**
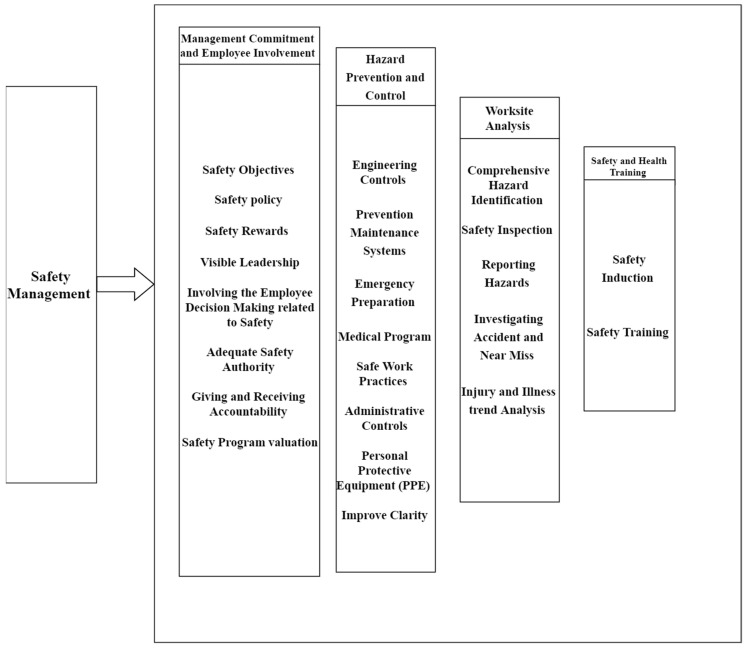
Safety program implementation elements.

This study’s EFA analysis singles out the significant (contributing) elements that affect safety implementation in the Iraq building industry (Figure 3).

The finding of this study can be used to increase safety performance by explaining the importance of safety program elements and the significant relationships between elements to improve these elements’ effectiveness. Furthermore, the findings may be used to guide management when developing or implementing a new safety program.

Regarding the previous discussion, there is a linkage between all four components. Top management should provide and support a successful safety program, sufficient resources to deal effectively with violence, and motivation to ensure the involvement of employees in safety management. Subsequently, the collaboration between workplace staff is implemented among the first stages in enhancing the identification and assessment of hazards; this step is a foundation for implementing an effective safety program. After completing an effective worksite analysis, the staff should take effective steps to prevent and control the identified hazards. All the above components depend on awareness. The provision of proactive and sufficient safety training can increase staff member awareness. This study approved the importance of training and communication in terms of their effects on safety awareness.

## 5. Conclusions

Impecunious safety performance is a global issue in the construction sector. The developed countries have demonstrated significant efforts to improve safety in the industry, and their efforts have resulted in significant progress and safety performance improvements. Developing countries, however, still lag behind because economic priorities tend to be their main focus. This research focuses on the Iraqi construction industry, where safety culture is poor and safety research is still scarce. Particularly, this research focuses on identifying the safety program elements, which are significant because the program’s successful implementation is an important step to improve safety performance.

After conducting a review of the literature, interviews, and collecting data using a questionnaire, this research identified 25 important safety program elements. Using EFA, these elements are grouped into four dimensions: management commitment and employee involvement, worksite analysis, hazard prevention and control, and safety and health training. There is an interrelationship among these dimensions. Management commitment is a foundational element that supports efforts to implement a safety program and improve safety performance. For this commitment to be achieved, it is also paramount to involve employees and make them accountable for safety. Strong management commitment facilitates collaboration among employees in conducting worksite analysis in the form of identifying safety hazards and risks. The next step is to implement strategies to prevent and control hazards and risks. Finally, safety and health training improve employees’ safety awareness, skills, and knowledge, supporting hazard and risk management.

The research findings contribute to the body of knowledge on safety research in the Iraqi construction industry, a research area which has not been much investigated thus far. The findings also provide guidance to industry practitioners and governments in Iraq in terms of focusing on the key elements that are needed to improve safety programs in this context. The findings of this study can be used as a guide for the decision-makers and the top management in construction projects for a better understanding of safety program elements and an effective implementation. In addition, the study will encourage developing countries to consider the implementation of the safety program in order to achieve their overall worker safety success.

Although the present study has achieved its aim, there are a few recommendations that future studies could focus on. A study on the impact of safety program elements on overall project success by using empirical methods would be of strategic importance, while presenting a country-specific checklist for the successful implementation of health and safety programs, as recommended by [70], could also offer some practical solutions.

## Figures and Tables

**Table 1:** Safety program elements.

	Code	Elements	Sources
Management Commitment and Employee Involvement (MCEI)	MCEI.EF.1	Safety objectives	[32,34,35]
	MCEI.EF.2	Safety policy	[32,34,35]
	MCEI.EF.3	Safety rewards	[36]
	MCEI.EF.4	Safety committee	[37,38]
	MCEI.EF.5	Visible leadership	[39]
	MCEI.EF.6	Involving the employee in decision making related to safety	[39]
	MCEI.EF.7	Adequate safety authority	[40]
	MCEI.EF.8	Giving and receiving accountability	[41]
	MCEI.EF.9	Safety program evaluations	[19,40]
Worksite Analysis (WA)	WA.EF.1	Comprehensive hazard identification	[1]
	WA.EF.2	Safety inspection	[34]
	WA.EF.3	Reporting hazards	[16]
	WA.EF.4	Investigating accident and near misses	[42]
	WA.EF.5	Injury and illness trend analysis	[41]
Hazard Prevention and Control	HPC.EF.1	Engineering controls	[1,41]
	HPC.EF.2	Preventive maintenance systems	[41,43]
	HPC.EF.3	Emergency preparation	[40,41]
	HPC.EF.4	Medical program	[1,43]
	HPC.EF.5	Safe work practices	[44]
	HPC.EF.6	Administrative controls	[43]
	HPC.EF.7	Personal protective equipment (PPE)	[1]
	HPC.EF.8	PPE hazard assessment and training	[43]
	HPC.EF.9	Improving clarity	[16,44]
Safety and Health Training	SHT.EF.1	Safety induction	[45]
	SHT.EF.2	Safety training	[46]

**Table 2:** The interviewees’ profile.

No	Position	Education Level	Experience (Years)	Sector	Organization Function
1	Director	BSc	30	Private sector	Contractor
2	Project manager	PhD	28	Government	Client/Developer
3	Site engineer	MSc	20	Government	Contractor
4	Senior manager	BSc	24	Private sector	Client/Developer
5	Consultant	PhD	40	Independent Consultant	Consultant
6	Senior manager	PhD	30	Independent Consultant	Consultant
7	Project manager	PhD	35	Independent Consultant	Consultant
8	Site engineer	MSc	15	Government	Client/Developer
9	Director	PhD	28	Independent Consultant	Consultant
10	Site engineer	MSc	12	Independent Consultant	Consultant
11	Site engineer	BSc	8	Private sector	Client/Developer
12	Director	MSc	25	Independent Consultant	Consultant
13	Project manager	PhD	22	Private sector	Contractor
14	Senior manager	MSc	15	Private sector	Contractor
15	Site engineer	BSc	10	Government	Client/Developer
16	Consultant	MSc	25	Independent Consultant	Consultant

**Table 3:** The demographic characteristics.

Variable	Level	Frequency	Percent
Gender	Male	130	86.7
	Female	20	13.3
Experience	5 years or less	23	15.3
	6 to 10 years	44	29.3
	11 to 15 years	48	32
	16 to20 years	19	12.7
	More than 20 years	16	10.7
Present organization	5 years or less	26	17.3
	6 to 10 years	49	32.7
	11 to 15 years	46	30.7
	16 to20 years	14	9.3
	More than 20 years	15	10
Current position in your organization	Director	8	5.3
	Senior manager	15	10
	Project manager	34	22.7
	Site engineer	63	42
	Other manager position	30	20
Education	Diploma	12	8
	Bachelor	72	48
	Master	53	35.3
	PhD	13	8.7
Organization Type	Public	92	61.3
	Private	58	38.7
Organization function	Client/developer	67	44.7
	Consultant	39	26
	Contractor	44	29.3

**Table 4:** The communalities of 25 safety program elements.

Item	Elements	Communalities	Item	Elements	Communalities
MCEI.EF.1	Safety objectives	0.573	WA.EF.1	Comprehensive hazard identification	0.559
MCEI.EF.2	Safety policy	0.720	HPC.EF.1	Engineering controls	0.673
MCEI.EF.3	Safety rewards	0.698	HPC.EF.2	Prevention maintenance systems	0.726
MCEI.EF.4	Safety committee	0.269	HPC.EF.3	Emergency preparation	0.627
MCEI.EF.5	Visible leadership	0.636	HPC.EF.4	Medical program	0.481
MCEI.EF.6	Involving the employee in decision making related to safety	0.646	HPC.EF.5	Safe work practices	0.762
MCEI.EF.7	Adequate safety authority	0.536	HPC.EF.6	Administration controls	0.712
MCEI.EF.8	Giving and receiving accountability	0.517	HPC.EF.7	Personal protective equipment (PPE)	0.806
MCEI.EF.9	Safety program evaluation	0.518	HPC.EF.8	PPE hazard assessment and training	0.611
WA.EF.5	Injury and illness trend analysis	0.523	HPC.EF.9	Improve clarity	0.567
WA.EF.2	Safety inspection	0.837	SHT.EF.1	Safety induction	0.816
WA.EF.3	Reporting hazards	0.795	SHT.EF.2	Safety training	0.800
WA.EF.4	Investigating accident and near misses	0.726			

**Table 5:** Total variance and the Eigenvalues.

	Component			
	1	2	3	4
Eigenvalues	5.155	4.462	4.356	2.162
% of Variance	20.622	17.85	17.424	8.648

**Table 6:** Reliability values.

Group Name	Reliability
MCEI	0.856
WA	0.886
HPC	0.916
SHT	0.868

## Data Availability

The data presented in this study are available on request from the corresponding author. The data are not publicly available due to privacy and confidentiality issues.
